# TRANscranial direct current stimulation for FOcal Refractory epilepsy in mitochondrial disease (TRANSFORM): delayed-start, randomised, double-blinded, placebo-controlled study

**DOI:** 10.1186/s12883-024-03907-6

**Published:** 2024-10-22

**Authors:** Katrin A. Bangel, Albert Z. Lim, Alasdair Blain, Yi Shiau Ng, Amy Winder, Joseph Bulmer, Grainne Gorman, Mark Baker, Robert McFarland

**Affiliations:** 1 Department of Northern Medical Physics & Clinical Engineering, Newcastle Hospitals NHS Foundation Trust, Newcastle upon Tyne, UK; 2 Translational and Clinical Research Institute, Faculty of Medical Sciences, Newcastle University, Newcastle upon Tyne, UK; 3 Wellcome Centre for Mitochondrial Research, Translational and Clinical Research Institute, Faculty of Medical Sciences, Newcastle University, Newcastle upon Tyne, UK; 4 Department of Paediatric Neurology, The Great North Children’s Hospital, Royal Victoria Infirmary, Newcastle upon Tyne, UK; 5 Directorate of Neurosciences, Royal Victoria Infirmary, Newcastle upon Tyne Hospitals NHS Foundation Trust, Newcastle upon Tyne, UK; 6 NHS Highly Specialised Service for Rare Mitochondrial Disorders of Adults and Children, Newcastle upon Tyne Hospitals NHS Foundation Trust, Newcastle upon Tyne, UK; 7 Department of Clinical Neurophysiology, Royal Victoria Infirmary, Newcastle upon Tyne, UK

**Keywords:** Mitochondrial disease, Mitochondrial epilepsy, Pharmacoresistant epilepsy, Delayed-start study design, Refractory focal seizures, Transcranial direct current stimulation (tDCS), DC stimulation, Cathodal neuromodulation

## Abstract

**Background:**

Focal epilepsy is common in children and adults with mitochondrial disease. Seizures are often refractory to pharmacological treatment and, in this patient group, frequently evolve to refractory focal *status epilepticus* (also known as *epilepsia partialis continua*). Where this occurs, the long-term prognosis is poor. Transcranial DC stimulation (tDCS) is a promising, non-invasive, adjunctive treatment alternative to common surgical procedures. Limited recruitment of study participants with this rare disease and the ethical challenges of administering a treatment to one group and not another, while maintaining strict methodological rigour can pose challenges to the design of a clinical study.

**Method:**

We designed the first delayed start, double-blinded, sham-controlled study to evaluate the efficacy of tDCS as an adjunctive treatment for focal epilepsy. We will include participants with a genetically confirmed diagnosis of mitochondrial disease with drug-resistant focal epilepsy aged ≥ 2 years, aiming to collect 30 episodes of focal *status epilepticus*, each treated for a maximum period of 14 days. The early start intervention arm will receive tDCS from day 1. The delayed start intervention arm will receive sham stimulation until crossover on day 3. Our primary endpoint is a greater than 50% reduction from baseline (on day 0) in seizure frequency assessed by 3x daily reporting, accelerometery, and video monitoring. Changes in the underlying epileptogenic focus within the brain related to the tDCS intervention will be assessed by magnetic resonance imaging (MRI) and/or electroencephalography (EEG).

**Discussion:**

Study results in favour of treatment efficacy would support development of tDCS into a mainstream treatment option for focal epileptic seizures related to mitochondrial disease.

**Trials registration:**

ISRCTN: 18,241,112; registered on 16/11/2021.

## Background

### Study rationale

The estimated prevalence of mitochondrial disorders is greater than 1 in 4300 [1, 2] and epileptic seizures are reported in about 20–60% of individuals with the diseas*e* [3–10]. Seizures are often refractory to treatment with antiepileptic drugs and neuronal dysfunction resulting from the underlying mitochondrial disease is thought to play a significant role not only in initiating but maintaining high levels of seizure activity. Diagnosis is guided by the clinical presentation, including lactic acidosis, refractory seizures, myoclonic seizures, neurodevelopmental delay or regression, stroke-like episodes or multiorgan dysfunction [11]. The prognosis for refractory seizure disorders due to mitochondrial disease is extremely poor and frequently associated with neurodegeneration [12, 13]. Whether increasing seizure activity contributes to neurodegeneration or is simply a consequence thereof is unclear. If the former, to improve quality of life and prognosis, new therapeutic approaches for suppressing seizure activity should be a priority.

Transcranial direct current stimulation (tDCS) non-invasively modulates cortical excitability through weak direct currents (typically 2 mA) applied through the scalp. In contrast to other electrical stimulation procedures, tDCS is significantly less invasive. Vagus nerve stimulation and deep brain stimulation typically involve invasive surgery, which carries risks including side effects of general anaesthesia, brain haemorrhage, infection, and accidental damage to other brain areas during device-implantation. In the long term, tDCS is potentially more cost-effective than taking multiple AEDs and inpatient (intensive care unit) treatment. Some AEDs require regular blood monitoring, which necessitates frequent hospital appointments. Intensive care admission and surgical procedures involve prolonged inpatient hospital admission. Hospital visits, both expected and unexpected, escalate costs (financial and non-financial) on caregivers (parents, siblings, partners, and families), even more so for children of school age.

Cathodal tDCS is thought to reduce the probability of sodium and calcium channel opening and thus the probability of action potential generation and seizure propagation. In addition, via a process of Long-Term Depression (LTD), a change in synaptic strength can be initiated that ultimately leads to structural long-term changes within the neuronal network [14, 15]. Several studies have provided evidence for the efficacy of tDCS in reducing the frequency of clinical seizures in people with refractory focal epilepsy [16–20]. Direct current stimulation may therefore control seizures by diverse mechanisms from reducing neural connectivity within epileptic networks and thus the probability of seizure propagation outside the epileptogenic zone, to reducing the energetic demands on mitochondria by indirect effects on spiking activity and possibly by direct effects on glia [21] and intracellular bioenergetics [22–24]; tDCS is thus a promising non-invasive, adjunctive treatment option for refractory epilepsy.

### Aims, objectives, and outcomes

This study aims to evaluate whether tDCS can serve as an effective adjunctive treatment to reduce focal epilepsy in people with mitochondrial disease. Reducing the number of seizures is a primary goal of treatment, making it a highly relevant outcome for both clinicians and patients. Seizure reduction is a commonly used and well-accepted metric in epilepsy research which can be objectively measured and is responsive to treatment, making it suitable for assessing the efficacy of tDCS. Sham stimulation will be used as a comparator to determine the true efficacy of the tDCS treatment by providing a baseline against which the effects of the active treatment can be measured.

As the *primary goal* we will assess the efficacy of tDCS, as an adjunctive treatment, in reducing the number of focal epileptic seizures experienced by children and adults with genetically confirmed mitochondrial disease.

The *primary endpoint* is a greater than 50% reduction in seizure frequency (number and duration of seizures, jerks/ min) from baseline (on day 0), based on the European Medicines Agency guideline of 26 July 2018 CHMP/EWP/566/98 Rev. 3 [25]. This will be assessed through daily reporting from specialists, nurses, research staff, relatives, and carers by means of a seizure diary, while accelerometery and video monitoring will corroborate seizure counts.

*Secondary objectives* will be fourfold:

1. We will assess seizure freedom (i.e., resolution of the focal epileptic event) after tDCS.
2. Magnetic resonance imaging (MRI) and electroencephalography (EEG) will be used to determine whether tDCS treatment results in clinically evident changes in the epileptogenic focus.
3. Finally, we will evaluate tDCS as a mainstream treatment option for focal epileptic seizures related to mitochondrial disease.

*Secondary endpoints* include:

1. Seizure freedom assessed by 3x daily seizure diary, accelerometery, video monitoring, and an end-of study EEG.
2. discontinuation of the study due to adverse events;
3. assessment of side effects by seizure diary;
4. and clinically significant improvement in neurophysiology and/or neuroradiology findings against expected natural history.

## Methods/ design

### Study design and participant management

This study follows a delayed-start, randomised, double-blind, sham-controlled design.

### Study setting

This single-site study will take place within the Wellcome Centre for Mitochondrial Research at Newcastle University, in collaboration with the NHS Highly Specialised Service for Rare Mitochondrial Disorders. All study assessments will be conducted in an adult or paediatric neurology ward at a tertiary care centre. Treatment sessions will be delivered on Newcastle-upon-Tyne Hospitals premises, unless the participant’s situation requires otherwise.

### Eligibility criteria

Adult and paediatric patients aged ≥ 2 years with a genetically confirmed diagnosis of mitochondrial disease and drug-resistant focal epilepsy as well as anatomically relevant changes related to focal seizures defined by neuroimaging and/or scalp EEG will be considered for inclusion. Drug-resistant focal epilepsy is defined by the International League Against Epilepsy (ILAE) as failure of two antiepileptic drugs to achieve sustained seizure freedom. Participants need to be able to undergo all study assessments and investigations in the opinion of the recruiting investigator in agreement with the participant/parents/legal guardian.

Participants will be excluded if they are aged < 2 years, have metallic or electronic implants, or have undergone other neurosurgical intervention (e.g. craniotomy) that typically preclude MRI scanning and/or tDCS treatment. People with other co-existing epileptic comorbidities e.g., brain tumour, traumatic brain injury, cortical dysplasia, or any other known uncontrolled medical problems that, in the opinion of the investigator, would preclude participation in the study will be excluded as well.

### Recruitment

This study will involve the assessment of at least 30 episodes of focal *status epilepticus* in participants with a genetically confirmed diagnosis of mitochondrial disease. This will either equate to the recruitment of 30 inpatient participants each presenting with one episode of focal *status epilepticus*, or fewer than 30 participants with individual participants contributing more than one episode of focal *status epilepticus* (provided each event per participant has a different focal location). If participants participate multiple times, they will be re-consented for each new focal event. If participants recruited into the study were already to be inpatients due to an acute episode of focal *status epilepticus* that cannot be resolved with antiepileptic drugs they will remain as such for the duration of their participation.

We host the largest UK mitochondrial disease patient cohort with over 1800 patients. Each patient on this cohort has actively consented to participate in research. This cohort has facilitated our robust feasibility assessment to establish that the time to target patient recruitment is attainable in the proposed timeline.

### Consent

All participants will be recruited by a qualified and delegated member of the research team trained in Good Clinical Practice (GCP). Consent to enter the study will be sought from each participant only after a full explanation has been given, an information pack offered, and time allowed for consideration. Signed consent will be obtained from participants before any study specific assessment or investigation.

Participants, or their carer/parent will be approached about this study early during their stay by a member of the research team and receive the study information pack. This will either be in person or remotely via video conferencing. Hence, they will have sufficient time to have all their questions addressed by the study team to decide whether to participate. They will have the opportunity to discuss the study and ask questions at any stage of the procedure. The information leaflets have been reviewed by several parents/carers of children with mitochondrial disease. The overall feedback has been positive, and the material was described as noticeably clear with enough information needed to make an informed choice. A copy of the signed informed consent form will be provided to the participant and a copy filed in the participant’s hospital records. The original signed consent form will be stored in the Investigator Site File.

Consent to ongoing participation in the study will be verbally re-confirmed with the participant and documented in their medical record at each subsequent assessment point (e.g., new focal epileptic event). Participants have the right to refuse to participate without giving reasons and without prejudicing further treatment. Documents will be translated to other languages if required. An interpretation service is available in the NHS Trust should this be requested. However, if the parent/guardian is unable to fully understand the study and cannot provide informed consent they would not be eligible to take part.

Participants aged 16 years or over with the capacity to provide consent, will be consented as adults and asked to complete their own informed consent form. For participants aged under 16 years, consent will be obtained on behalf of the participant from their parent or a person with parental responsibility. Where appropriate, written assent will be obtained from the child. If a participant has their 16th birthday whilst participating in the study, they will be re-consented as an adult. If they lack capacity to provide consent, a consultee will be asked to provide a consultee declaration on the participant’s behalf. Additional consent includes permission to obtain information from medical records (until six months after study end), to obtain accelerometer data, and to obtain optional short video recordings. No biological specimens will be collected.

### Participant timeline

**Fig. 1:**
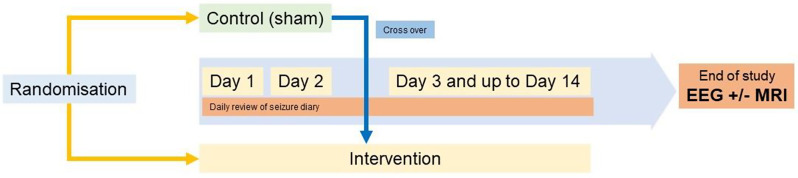
Overview of study events. Please note that patients will only receive the MRI procedure if considered necessary from a clinical point of view as part of the patients’ regular care

After consenting (legal guardian/carer) and assenting (for children who are capable), all participants will be allocated a unique study number. Several screening procedures will be performed including – basic demographics, complete medical history with physical examination and vital signs, and pre-treatment baseline assessment of seizure frequency (see Table 1). Participants who complete screening and meet all study requirements, will be randomised, and enter the treatment period on Day 1 (see Fig. 1).

**Table 1:** Schedule of events

Procedure	Screening	Intervention	Follow-up	Frequency	Duration (mins)	Performed by:
Informed Consent	**X**			1x	45	Study team
Demographics	**X**			1x	5	Study team
Medical History	**X**			1x	15	Study team
Physical Assessment	**X**			1x	5	Study team
Confirmation of Eligibility	**X**			1x	15	Study team
tDCS		**X**		1x daily for max. 14 days	30	Study team (or trained carer)
Seizure diary	**X**	**X**		3x daily for max. 15 days	varies	Patient/carer/legal guardian
Video monitoring of seizure area (if consented)	**X**	**X**		1x daily for max. 15 days	5	Patient/carer/legal guardian
Accelerometery recordings	**X**	**X**		2x	Continuous	Patient/legal guardian/ or study team
Adverse Event Reporting	**X**	**X**	**X**	Max. 15 days	20	Study team
End-of-study EEG			**X**	1x	30	Study team

*Note* Administration of tDCS will end with resolution of the focal epileptic event (i.e. seizure freedom), or after 14 days, whichever happens earlier

## Intervention procedures

### Randomization and intervention protocol

At baseline, participants will be randomly assigned to receive either sham stimulation (delayed-start group) or active tDCS (early-start group). The placebo group will receive double-blinded sham procedure for 2 days. On Day 3, all participants will receive the active tDCS intervention as an adjunctive treatment for up to day 14. The allocated intervention will not be modified.

At baseline, participants will be randomly assigned to one of two arms for each focal event:

In the **Early Start Group** participants will receive 20 min of active tDCS daily from the start of the intervention period (day 1) for up to 14 days. By default, each active stimulation treatment session starts with an automated ramp up of current to 2 mA over 15 s to keep participants blind to the intervention. For the early start group tDCS then continues for 20 min until it ramps down.

In the **Delayed Start Group** participants will receive sham-stimulation/placebo treatment on day 1 and day 2 of the intervention followed by up to 12 additional days of active tDCS (1x daily for 20 min). Like active tDCS, sham tDCS starts with an automated ramp up of current (from 0 to 2 mA over 15 s) to mimic the sensations observed with active tDCS and to keep participants blind to the intervention. For the sham tDCS the current stops after the 15s ramp up. This sham stimulation protocol is well supported by current literature [16, 17].

### Intervention administration and assessment procedures

Participants will undergo the following interventions and assessments as part of the study:

1. **Cathodal transcranial direct current stimulation (tDCS) at 2 mA**: The treatment will be administered 1x daily (every 24 h) for a period of up to 14 days. Participants will either receive active tDCS from day 1 (early-start group) or cross over from sham treatment to active tDCS on day 3 (delayed-start sham group). Neither the participant nor the treating physicians will be aware of the participants’ randomisation allocation. The scalp location for applying tDCS will be defined by the location of the epileptogenic focus responsible for the clinical seizures. Administration of tDCS will end with resolution of the focal epileptic event (i.e., seizure freedom), or after 14 days (whichever is earlier).
2. Participants, their carers, or hospital staff will complete a seizure diary three times daily throughout the intervention period. Throughout the entire study, from Day 1 up to Day 14, seizure frequency (including onset time and duration of seizures) will be documented three times daily. This diary may also record seizure count, seizure type, frequency of twitches, potential medication changes and/or possible seizure triggers.
3. Participants will undergo a study-specific **end-of treatment standard clinical EEG** for each focal event.
4. **Short videos**, lasting 1–2 min, will be recorded daily throughout the treatment period to document seizure frequency in the affected body areas. Parents/carers will capture these videos following detailed instructions provided to them. Due to limited resources, it will not be possible to provide equipment on which to record videos at home therefore the ability to record videos at home will depend upon the equipment available. Participants, or their parents/carers, may opt out of video recording entirely or choose to have recordings done exclusively in the clinic.
5. **Accelerometery (continuous throughout treatment period)**: To monitor seizure activity we will measure 3D motion data from the areas of the body that are affected by the participants’ motor seizures one day prior to the start and during the 14-day treatment period. The participant will wear accelerometers that, depending on the body parts that are affected by seizures, can either be fastened around the wrist or securely taped to the body. We will collect 3D movement, vibrations, and orientation changes to verify seizure activity. The device is waterproof and should be worn continuously during the treatment period. The device is CE safety mark approved and compliant with the Directive 2014/30/EU. Consent to wearing the device is optional.

Pre-study EEGs and brain MRIs will be acquired as part of the participants’ standard care for diagnosis and localisation of the epileptic focus. MRI and EEG safety screening questionnaires will have been completed before each exam as part of participants’ standard care. In the absence of an MRI lesion, the tDCS target will be determined based on standard clinical scalp EEG. These procedures will not be part of the study, but neuroimaging results will be considered for treatment-related decisions (e.g., specification of the tDCS target) and treatment evaluation. Only the ‘end of study’ standard clinical EEG will be collected as part of the study. Participants will receive a post-intervention MRI procedure if considered necessary from a clinical perspective as part of the patients’ routine care. Those MRI scans are not a standard research assessment related to this study but will be included in the data analysis when consent has been explicitly obtained for that purpose.

### Intervention procedure

Participants will be seated and their skin on the scalp around the stimulation areas will be inspected to ensure it is healthy and intact. Any metallic objects (hairpins, glasses, jewellery) near the electrodes will be removed. The tDCS system will be visually inspected, including checking the battery status. According to the participant’s head circumference a suitably sized neoprene cap with chinstraps will be selected. Based on the participants’ seizure location, electrodes will be attached to the cap. A medical professional will determine electrode location individually for each focal event, based on the MRI, EEG, and patient history. Electrode location is determined prior to the treatment and remains the same throughout the study for each focal event. Electrodes will be clipped onto the pre-determined position. As a rule, the cathode will be located over the seizure site and the anode maximally distant to the seizure site. To avoid mix-ups of electrodes, we customize caps with marked insertion holes for the electrodes at each stimulation location, we provide in-person training, customised written instructions and remote video guidance. The cap will be placed on the participants head with a conductive medium (15 ml of NaCL 0.9%) applied between electrode and scalp. The device will be placed safely (away from liquids, no tripping hazards etc.). Spillage of the conductive medium will be avoided by removing any excess electrolyte before application to the head. We will control the position of electrodes and caps before treatment starts to keep the optimal treatment constant over the entire study. Stimulation will only start if the impedance is below 15 kΩ. Stimulation will automatically be paused if impedance increases above this threshold. The device times the treatment and automatically stops current flow after 20 min. Throughout the therapy session, impedance and current flow will be monitored.

### Study device

The tDCS device supplies current at a constant strength. The Sooma tDCS, manufactured by Sooma Oy, Helsinki, Finland will be used to deliver the direct current stimulation. As part of this system, two electrode cups with hydrogel stimulation pads inserted will be used to deliver the current. Pads will be soaked in 15 ml saline solution causing it to expand and form a soft and uniform stimulation surface. The neoprene cap will be used to keep the electrodes in place. The device is designed for at-home treatment, offering ease of use and portability (please refer to tDCS setup and stimulation section for information about the safe use of the device). The treatments can be self-administered by the participant/legal guardian/carer at home, under the remote supervision of a medical professional.

### Strategies to improve adherence to the intervention

Adherence to the intervention is facilitated by close monitoring of the participant by the study team. If patients receive treatment at home, they will be monitored remotely.

### Withdrawal and relevant concomitant care permitted during the study

Standard care for participants continues throughout the study. No intervention is prohibited. Participants have the right to withdraw from the study at any time without having to give a reason. The investigator may withdraw a participant from the study at any time if the investigator considers it necessary. As per consent, if participants are withdrawn from the study, the information already obtained will be kept. Patients will be able to continue their regular treatment. If they wish, patients will be able to continue their (daily) tDCS treatment according to the study treatment plan, even if they withdraw from the study. In that case, no further study data will be collected after withdrawal.

### Provisions for post-study care

Standard care will continue within the UK National Health Service (NHS) during and after the study.

### Intervention allocation

The experimental unit for this study will be a single patient episode (rather than the patient) identified by an event ID, since one patient can have two or more separate episodes of focal *status epilepticus* over the course of the study. Participant event/episode IDs will be randomly assigned to groups in a 1:1 ratio using block randomisation (6 blocks) by the sealed envelope software [26]. Based on the event ID the dedicated team member will be able to infer the study arm that determines the device setup for the first two days, i.e. active (early start) vs. sham (delayed start).

TDCS device marked as #1 will be set up individually for each experimental event for day 1 & 2 to either sham or active treatment with the aid of a special tool and instructional video. A second device marked as #2 will permanently be set up for active treatment and used for the remaining sessions (3 up to 14). One member of the team, not involved in the delivery of tDCS or data analysis, will be responsible for the randomization process, and sham/active device assignment and delivery. The random group assignment will be kept secret in a password protected file only accessible to the responsible team member. All remaining team members and all participants and relatives will be unaware to which treatment arm the participant is assigned. A record will be kept confirming when and by whom the randomisation code was requested and provided.

### Blinding

The study team, as well as the participant, parents, carers, and any consultee, will be blinded to the randomisation (double blinding). The team member who sets the randomisation will not be involved in any other study-related activities.

Block randomisation prevents accidental unblinding affecting the blinding of remaining study events. Unblinding at the end of study will happen after the statistical analysis plan is finalised. Early withdrawal based on adverse events (AE) may require unblinding.

## Assessment and collection of outcomes

### Pre-intervention screening

Following informed consent, participants will undergo the following screening assessments to confirm eligibility for the study:

- Collection of basic demographic information (name, address, date of birth, NHS number).
- Collection of full medical history.
- Physical examination and vital signs (height, weight, heart rate, respiration rate, blood pressure and head circumference).
- Baseline seizure frequency assessment through seizure diary and video assessment on day 0.

These assessments will be documented in the participant’s hospital records.

### Intervention period

Participants who are confirmed to be suitable for continued participation in the study will then undergo the following assessments/activities:

- Randomisation.
- tDCS for a period of 20 min once a day, for a period of up to 14 days.
- Completion of a seizure diary/collection of seizure events daily over the tDCS treatment period.
- 1–2 min of daily video recordings of areas of the body affected by seizures (if consented).
- Continuous accelerometery measurements (in consented).

### Post-intervention

Following the tDCS treatment period, participants will undergo the following assessment:

- End of study EEG (obtained the day after the last intervention day).
- Further follow up through regular clinical care. For six months data will be collected from the patient’s medical record.

Unless the study is prematurely discontinued, the end of the study will be the date of the last visit of the last participant and/or the completion of all follow-up monitoring.

## Data management and confidentiality

### Data collection tools and source documentation identification

Completed study consent forms will be held in the Investigator Site File. Copies will be held in the participant medical records. Source data for this study will consist of annotations in the participant medical records, patient diaries, video recordings, accelerometery data, and data from EEG and MRI exams. Completed researcher/clinician assessment tools will be stored in the Investigator Site File.

Participants will be identified on any assessment tools via their unique study ID number rather than by name. Data will be transcribed from the source data directly onto the study database. Participants and study events will be identified on the database via the unique study ID number (i.e., the study database will be classed as containing pseudonymised data only).

As part of the study, short videos (1–2 min) of participants’ areas of the body affected by their seizures will be captured (either in clinic or by parents/carers at the home). These videos will be transferred onto secure NHS servers and pseudonymised (i.e. by blurring out facial features) prior to transfer onto Newcastle University systems for long-term storage/analysis. Measures will be taken to ensure that the identifiable images (i.e. prior to pseudonymization) are transferred securely. Participants will be provided with information about how the data from these videos will be managed and stored as part of the informed consent process. This is an optional assessment; participants will be advised of this during recruitment and of the fact that they can still participate in the study without video capture.

All efforts will be made to ensure that the data provided in the source documents is as complete as possible. Regular review of data completeness and regular data cleaning activities will be undertaken. These activities may include telephoning participants to obtain missing information. Any activities which involve contacting study participants will be conducted by delegated members of the site team who are known to the participant (i.e. study research nurse).

### Data handling and record keeping

The study will comply with all relevant data protection laws. Data entry will be performed by a member of the research team at the site. The study database will include Excel spreadsheets and files in .csv format (for EEG) and DICOM format (for MRI) which will be read with other appropriate software approved by the Sponsor. The database will be held on secure servers within Newcastle University. Access to the area of the server containing the study database will be restricted to research team members only. Permission to access the study database will be issued by the Chief Investigator. The link between each participant unique study ID number and their name will be via the study recruitment log, which will be held in the Investigator Site File (ISF), an electronic version of this log may also be held securely on NHS computer systems.

The ISF will be held in a secure area within Newcastle Hospitals with access restricted to the study team only. Following completion of the study, fully anonymised sets of raw data may be made available for 3rd party research purposes with the appropriate data transfer procedures.

### Access to data and dissemination of results

Direct access to study data including source data contained in the participant medical notes and personal identifiable data contained in the ISF will be granted to authorised representatives of the Sponsor, Newcastle University or regulatory authorities for the purposes of monitoring, audit or inspection. Consent for this will be obtained from participants during recruitment.

Study participants will be advised in the participant information sheet that they can contact the study team to request a summary of the research results once the study is completed. The results of this study will be disseminated via peer-reviewed scientific journals, conference presentations and other publications.

### Archiving

Archiving will be authorised by the study sponsor following submission of the end-of-study reports to the REC and funder. Essential documents will be archived for a period defined by the study sponsor and archiving will be according to sponsor processes and procedures. Destruction of essential documents following the required period of archiving will require sponsor authorisation. Pseudonymised study data held on the study database will be retained on Newcastle University servers for 20 years following the end of study and used for further analysis. Long term storage of data will be in accordance with the Newcastle Joint Research Office policy for archiving using the approved archiving facility. Arrangements to ensure data security will be in accordance with NuTH Trust policies and procedures.

## Statistical methods

### Statistical analyses

The data will be tabulated using descriptive statistics. Mean and standard deviation will be reported for continuous data. Median and range will also be reported for skewed data. Categorical data will be described using percentages and frequencies.

The primary analysis of the binary outcome of a 50% reduction in epilepsy frequency (number and duration of seizures, jerks/min) from baseline (counted on day 0) will be completed by applying a generalised estimating equation to thus obtain robust standard errors and test whether the tDCS group improves more rapidly than the sham group.

Further analysis will consider a more complex model incorporating random slopes where appropriate. Sensitivity analysis for missing data will also be investigated. Other outcome data will be analysed as appropriate using a mixed effects model for continuous data and a generalised estimating equation for binary, categorical or count data with appropriate distribution and link function.

### Interim analyses

No interim analyses are planned.

#### Target difference and sample size

Assuming a minimum of one assessment per patient event per day, each event will contribute 14 data points over 14 days of treatment (tDCS or sham). Further assuming no association between days of treatment and reduction in epilepsy events, the outcome data are treated as clustered data for the purposes of the power calculation. Assuming 5% type I error, 90% power, and 10% intra-patient correlation, 28 patient events will be required over 14 days of treatment to detect an improvement of 20% in events that achieved 50% reduction in seizure frequency. The sample size calculation also assumes that only 10% of the events in the placebo group will achieve 50% reduction in epilepsy frequency from baseline (on day 0). To account for drop-out, 30 patient events will be required for this study. The patient events will be allocated equally to active and sham tDCS groups. These calculations were determined based on clinical significance, patient perspectives, historical data, statistical considerations, and regulatory guidelines [25].

#### Oversight and monitoring

##### Composition of the coordinating centre and study steering committee

As part of the pre-study risk assessment the ethics committee identified that a study steering committee is not required for this non-CTIMP clinical study of a CE marked device for an off-label indication.

##### Composition of the data monitoring committee, its role and reporting structure

No formal data monitoring committee will be convened. This study is classed as non-CTIMP and the ethics committee approved that the study treatment is not predicted to cause high morbidity or mortality or is not associated with unknown or uncertain risks. Instead, interim safety and efficacy will be reviewed on a regular basis. An annual progress report will be submitted each year to the REC by the CI until the end of the study. This report will be submitted within 30 days of the anniversary date on which the original favourable ethical opinion was granted.

### Adverse event (AE) reporting and harms

AE reporting will be restricted to adverse events occurring during or resulting from the study intervention (tDCS) or study assessments. Such adverse events will be documented in the participant medical records recording causality and severity and will also be recorded on the study adverse event log held in the Investigator site file. AEs will be followed up until resolution or until stabilisation (if complete resolution is not anticipated). Any other adverse events/serious adverse events that occur will not be reported as AEs, including any events occurring during or related to the participant’s inpatient hospital stay. In the event of a Serious Adverse Event (SAE), the sponsor, The Newcastle upon Tyne Hospitals NHS Foundation Trust will be notified immediately by email (within 24 h of site awareness of the event). If complete information is not available or if a PI/Investigator cannot be obtained within 24 h, the SAE report should be submitted as incomplete in the first instance and the missing details provided at the earliest opportunity. SAEs will be followed up until resolution or until stabilisation (if complete resolution is not anticipated) and updated reports should be provided to the sponsor as required until resolution. All SAEs will be recorded in the participant medical notes and recorded on the study adverse event log held in the Investigator Site File (ISF)/ Trial Master File (TMF). Any SAE notification emails, including updated notifications, will be retained in the ISF and TMF. Any SAEs that are related to the study intervention/assessments and that are classified as unexpected will be reported to the Research Ethics Committee (REC) by the Sponsor and reported to other Sponsor departments as per Sponsor procedures. Any abnormal results or issues of concern identified during any study visit or during the analysis of the study samples will be documented and referred to the Principal Investigator for discussion with the participant’s routine clinical care team to decide about (dis-)continuation of the treatment.

### Frequency and plans for auditing study conduct

The study is monitored and audited by the sponsor. The CI will notify the REC of any adverse events or the early termination or end of study in accordance with the required timelines.

Any change to this protocol after approval has been given will be notified as an amendment to relevant parties.

## Discussion

### Study design, practical and operational challenges

Refractory focal epilepsy associated with a mitochondrial disorder, while rare, often has a poor prognosis. Here, we address the unique challenges encountered in designing a randomized, sham-controlled study within the context of rare diseases.

First, designing this randomised sham-controlled study has posed ethical challenges of administering a treatment to one group and not another while keeping high methodological stringency. Our team’s prior treatment of five patients with mitochondrial epilepsy, who had received cathodal tDCS on compassionate grounds, suggests that focal episodes may resolve within the first three days of stimulation [27]. Based on that, we adopted a delayed-start design. One group receives the treatment immediately (early-start), while the delayed-start group receives a non-active (sham) treatment for the first two days and then receives active treatment from day 3.

Secondly, recruiting patients with a condition classified as a rare disease who experience an acute event is logistically challenging and, in this case, requires active recruitment methods. Potential participants will be identified by their health care provider while patients are admitted to the hospital for an acute focal epileptic event that remains unresolved by antiepileptic drugs. Consequently, the timeframe of recruitment is unpredictable, and to complete recruitment within the 3-year study period, individual means of recruitment may be considered.

Third, for some participants admission to our premises could be disruptive e.g., if their symptoms can be managed externally. To not exclude any potential participants from entering the study, we consider remote treatment for those patients. In that case patients would be referred to our site for their tDCS treatment. After initial supervised device training, tDCS will be self-applied by patients at home or in their care setting, under the remote supervision of a medical professional. Depending on the participants’ circumstances, we will determine individually which option is least disruptive.

Fourth, patients exhibit various types of seizures, each requiring unique methods of recording and diagnosis. To ensure optimal daily monitoring of seizure activity, we maintain close monitoring of the patient, provide flexible seizure diary templates, and record relative values.

### General discussion and conclusions

tDCS is being recognized as a promising non-invasive, non-pharmacological, adjunctive treatment for refractory epilepsy due to its low-risk profile, low cost, and ease of use compared to other surgical neurostimulation techniques such as deep brain stimulation and vagus nerve stimulation [16]. Moreover, in many people with epilepsy, anti-epileptic drugs become less effective over time, leading to seizures that are refractory to medical therapy. Surgical removal of an epileptic focus is an invasive procedure that could potentially be postponed or even avoided by neuromodulation. Several studies involving patients with refractory focal epilepsy have shown evidence supporting the efficacy of tDCS in reducing the frequency of clinical seizures [16–20]. Based on 27 studies, including nine randomized controlled trials, cathodal tDCS was evaluated as safe and likely effective for seizure control in patients with drug-resistant focal epilepsy [28]. However, larger sham-controlled randomized studies are needed to further advance tDCS therapy in the management of epilepsy.

In conclusion, while existing studies provide evidence for the efficacy of tDCS in refractory epilepsy, its effectiveness as an adjunctive treatment in focal refractory epilepsy in mitochondrial disease remains inconclusive. Study findings from this delayed start, double-blind, sham-controlled study showing treatment efficacy would contribute to the development of tDCS as a mainstream treatment option for focal epileptic seizures associated with mitochondrial disease.

## Data Availability

No datasets were generated or analysed during the current study.

## Abbreviations

AE: Adverse Event
CE: Conformité Européenne (‘European conformity’)
CI: Chief Investigator
CRF: Case Report Form
CTIMP: Clinical Trial of Investigational Medicinal Product
DICOM: Digital Imaging and Communications in Medicine
EEG: Electroencephalogram
GCP: Good Clinical Practice
GP: General Practitioner
HRA: Health Research Authority
HTA: Human Tissue Authority
ICH: International Council for Harmonisation of Technical Requirements for Pharmaceuticals for Human Use
ICF: Informed Consent Form
ILAE: International League Against Epilepsy
ISF: Investigator Site File
LTD: Long-Term Depression
MHRA: Medicines & Healthcare Products Regulatory Agency
MRI: Magnetic Resonance Imaging
NHS: National Health Service
NICE: National Institute for Health and Care Excellence
NuTH: Newcastle upon Tyne Hospital Foundation Trust
PI: Principal Investigator
PIS: Participant Information Sheet
R&D: Research & Development
REC: Research Ethics Committee
SAE: Serious Adverse Event
SOP: Standard Operating Procedure
SUSAR: Suspected Unexpected Serious Adverse Reaction
TDCS: Transcranial direct current stimulation
TMF: Trial Master File
UK: United Kingdom
UKCA: UK Conformity Assessed
